# Revitalising Postgraduate Psychiatry Training in Myanmar Following the 2021 Military Coup

**DOI:** 10.1192/bjo.2026.11173

**Published:** 2026-06-30

**Authors:** Maung Oakarr, Yan Lin Aung, T Tina, Maung MaNaw, W Winnie

**Affiliations:** Myanmar Board of Psychiatry, Rangoon, Myanmar

## Abstract

**Aims:**

**Background/Rationale**

The military coup in Myanmar in February 2021 caused widespread disruption to medical education. Many psychiatry trainees and trainers joined the Civil Disobedience Movement (CDM), resulting in the suspension of formal postgraduate psychiatry training nationwide and posing a serious threat to the future psychiatric workforce. In response, Myanmar CDM psychiatrists, supported by the psychiatrist diaspora, developed an alternative pathway to sustain postgraduate psychiatry training in a conflict setting.

**Aims/Objectives**

To restore postgraduate psychiatry training while maintaining academic standards, clinical competency, and professional integrity through an innovative, digitally enabled, and internationally supported training model.

**Methods:**

Biweekly online Continuing Medical Education (CME) sessions commenced in March 2021. By September 2023, CDM psychiatrists collaborated with retired academics and diaspora psychiatrists from the United Kingdom, United States, and Australia to develop a formal curriculum. The programme was finalised in December 2023 and approved by the Federal Health Professional Council and Interim University Councils. An online Master of Medical Science (Psychiatry) programme was launched on 15 January 2024. The programme focused on clinical knowledge development through weekly Zoom lectures and Moodle-based learning, and clinical skills development through supervised practice, CME case discussions, and workplace-based assessments. Research training was deferred due to safety concerns.

**Results:**

Trainee engagement was monitored through assignment completion, participation in synchronous teaching, and completion of supervised clinical attachments. Ten trainees enrolled. Assignment completion reached 100%, participation in teaching was at least 75%, and clinical attachment completion exceeded 90%. Between April and May 2025, seven trainees completed final assessments, including multiple-choice examinations and virtual clinical examinations with live assessors and simulated patients. All seven trainees passed and were certified as qualified psychiatrists. A 360-degree evaluation demonstrated high satisfaction among trainees, supervisors, and assessors.

**Conclusion:**

Despite severe disruption, Myanmar’s CDM psychiatrists successfully revitalised postgraduate psychiatry training using a decentralised, digitally enabled, and internationally supported model. This experience demonstrates that high-quality postgraduate psychiatry education can be sustained in fragile and conflict-affected settings and offers a scalable framework for global mental health workforce development.

**Acknowledgements** **:** We acknowledge all Civil Disobedience Movement (CDM) mental health professionals in Myanmar and the Myanmar psychiatrist diaspora worldwide for their collective leadership, academic contribution, and unwavering commitment to sustaining postgraduate psychiatry training under conflict conditions.

